# Identification and Characterization of a *cis*-Regulatory Element for Zygotic Gene Expression in *Chlamydomonas reinhardtii*

**DOI:** 10.1534/g3.116.029181

**Published:** 2016-03-23

**Authors:** Takashi Hamaji, David Lopez, Matteo Pellegrini, James Umen

**Affiliations:** * Donald Danforth Plant Science Center, St. Louis, Missouri 63132; † Department of Molecular, Cell, and Developmental Biology, University of California, Los Angeles, California 90095

**Keywords:** *Chlamydomonas*, *cis*-regulatory element, fertilization, homeodomain protein, zygote

## Abstract

Upon fertilization *Chlamydomonas reinhardtii* zygotes undergo a program of differentiation into a diploid zygospore that is accompanied by transcription of hundreds of zygote-specific genes. We identified a distinct sequence motif we term a zygotic response element (ZYRE) that is highly enriched in promoter regions of *C*. *reinhardtii* early zygotic genes. A luciferase reporter assay was used to show that native ZYRE motifs within the promoter of zygotic gene *ZYS3* or intron of zygotic gene *DMT4* are necessary for zygotic induction. A synthetic luciferase reporter with a minimal promoter was used to show that ZYRE motifs introduced upstream are sufficient to confer zygotic upregulation, and that ZYRE-controlled zygotic transcription is dependent on the homeodomain transcription factor GSP1. We predict that ZYRE motifs will correspond to binding sites for the homeodomain proteins GSP1-GSM1 that heterodimerize and activate zygotic gene expression in early zygotes.

Changes in ploidy are fundamental transitions for eukaryotic cells undergoing sexual reproduction. Fertilization merges the contents of two gametic cells and initiates a zygotic development program that leads to formation of a new individual in diploid or sporophyte-dominant species or to recombination and meiosis in haploid or gametophyte-dominant species.

*Chlamydomonas reinhardtii* is a haploid unicellular green alga that reproduces mitotically (vegetative reproduction) under nutrient replete conditions. Nitrogen starvation triggers gametic differentiation into one of two mating types (*plus* and *minus*) that are governed by a single multigenic locus, *mt*, on Chromosome VI with two haplotypes, *mt+* and *mt−* (Goodenough *et al.* 2007; Nishimura 2010). Fusion of two *C*. *reinhardtii* gametes of opposite mating types mixes cytoplasm and organelles from two different strains and leads to nuclear fusion, selective degradation of organellar genomes (uniparental inheritance), and formation of a thick, environmentally resistant cell wall that protects the dormant zygospore. Initiation of zygotic development is triggered by formation and nuclear translocation of a heterodimeric KNOX/BELL-type homeodomain protein dyad GSM1/GSP1 whose subunits are expressed individually in *mt−* (GSM1) or *mt+* (GSP1) gametes (Kurvari *et al.* 1998; Zhao *et al.* 2001; Lee *et al.* 2008; Nishimura *et al.* 2012). Previous studies identified a limited number of zygotic genes (Ferris and Goodenough 1987; Uchida *et al.* 1993; Kuriyama *et al.* 1999; Ferris *et al.* 2002; Kubo *et al.* 2008), and to date, all zygotic genes that have been examined depend on GSP1-GSM1 for expression (Zhao *et al.* 2001; Lee *et al.* 2008; Nishimura *et al.* 2012). Moreover, ectopic expression of GSP1-GSM1 in gametes is sufficient to induce haploid zygotic gene expression and differentiation in haploids (Zhao *et al.* 2001; Lee *et al.* 2008). A possible regulatory motif for zygotic genes (CGtGACATGaCC) has been suggested based on a small subset of known zygotic genes (Lee *et al.* 2008). Additionally, Uchida *et al.* (2004) reported the identification of cAMP response elements in the promoter region of *ZYS3*, one of the most strongly induced zygotic genes, but to date, a comprehensive genome-wide search and functional analysis of zygotic gene regulation has not been described.

Recently, we performed a genome-wide transcriptome study that enabled the comprehensive identification of 627 early zygotic genes (Supplemental Material, Table S1; Lopez *et al.* 2015). Here we used this early zygotic gene set to search for enriched sequence motifs that play a role as *cis*-regulatory elements in controlling zygotic gene transcription. We identified a 9-mer motif YGACAYGAC that is unrelated to cAMP response elements and which was overrepresented in the upstream regions of zygotic genes. The motif was named ZYRE (zygotic response element) and its importance for activating zygotic gene expression was tested using luciferase reporter constructs. When the reporter was fused to the promoter of zygotic gene *ZYS3* luciferase expression was brought under zygotic control. When ZYRE elements were fused to a tubulin minimal promoter they also conferred zygotic expression. Furthermore, the ZYRE-controlled luciferase expression was not observed when the *mt*+ parent carried a *gsp1* mutation. These experiments show that the ZYRE element is necessary and sufficient to drive zygotic gene expression under the control of a key regulator of zygotic development, GSP1. We propose that ZYRE elements may be directly bound and regulated by the zygotic homeodomain transcription factor GSP1-GSM1 that is formed upon fertilization.

## Materials and Methods

### Strains and growth conditions

*C*. *reinhardtii* strains 21gr *mt*+ (CC-1690) and 6145c *mt*− (CC-1691) were used for transformation experiments, and mating tester strains R3 *mt*+ (CC-620) and CJU10 *mt*− (Umen and Goodenough 2001) were used for mating experiments as described below. The *gsp1 mt*+ mutant strain was generated from F1 progeny after crossing 21gr *mt*+ and *gsp1 mt*− strain LMJ.SG0182.003395 distributed by the Chlamydomonas Stock Center (www.chlamycollection.org) through the Chlamydomonas Library Project (https://www.chlamylibrary.org; Zhang *et al.* 2014; Li *et al.* 2016) following standard procedures (Harris 2008). Disruption of the *GSP1* gene in the mutant was confirmed by sequencing the insertion border on one side of the insertion cassette following the procedure described previously (Li *et al.* 2016) with the primers listed in Table S2. Amplification of regions distal to the unmapped insertion border were used to confirm the absence of large deletions in *GSP1* or adjacent genes (*GSP1* CDS: gsp01 and gsp02; *GSP1* cassette insertion: gsp01 and oMJ155; *GSP1* 3′-UTR: GSP1-3fwd and GSP1-3rev; bHLH protein encoding gene: 7_bHLH_F and 7_bHLH_R, used in Nishimura *et al.* 2012). Strains were grown in liquid TAP medium or on TAP 1.5% agar plates as previously described (De Hoff *et al.* 2013; Lopez *et al.* 2015). The fertilization rate (%) was scored as follows: 100 × (number of quadriflagellated cells)/[(number of unmated cells) + 2 × (number of quadriflagellated cells)].

### Zygotic motif identification

Gene models used for sequence retrieval were downloaded from Phytozome 10 (v5.5, DOE Joint Genome Institute; http://phytozome.jgi.doe.gov; Merchant *et al.* 2007; Blaby *et al.* 2014) using the BioMart platform (Smedley *et al.* 2009). Individual portions of genes [500 bp upstream of start codons for gene models, upstream regions to transcription start sites (TSSs), 5′-/3′-UTRs, whole gene models and coding sequences (CDSs)] were retrieved using BioMart. MEME (Multiple Expectation maximization for Motif Elicitation; Bailey and Elkan 1994) was used to find enriched motifs in the 500 bp regions upstream of CDSs for the 300 most highly upregulated zygotic genes identified in our earlier study (Lopez *et al.* 2015) with the background null hypothesis third order Markov model constructed from corresponding regions of all *C. reinhardtii* genes. FIMO (Find Individual Motif Occurrences; Grant *et al.* 2011) was used to count occurrences of the ZYRE motif in different sequence sets. Segmented FIMO frequencies were tested statistically using Student’s *t*-test to assess enrichment in zygotic genes *vs.* a background null model composed of sequences from all gene models.

### Vector construction

Luciferase reporter constructs were made as follows: The *HSP70A*/*RBCS2* promoter contained on a *Xba*I-*Xho*I fragment in *C. reinhardtii* reporter vector pHsp70A/RbcS2-cgLuc that includes a *loxP* site (Heitzer and Zschoernig 2007; Ruecker *et al.* 2008) was replaced with fragments containing different *cis*-regulatory elements (see below) that were amplified with primers containing *Xba*I and *Xho*I sites (or compatible sites for ligation). PCR fragments were cut with appropriate enzymes and ligated to *Xba*I-*Xho*I cut pHsp70A/RbcS2-cgLuc. Figure S1 describes sequences of transgenes used in the luciferase expression experiments.

Promoter-containing inserts were amplified using Phusion DNA polymerase in GC Buffer (New England Biolabs, Ipswich, MA); cycles: 98° for 2 min and 30 cycles of 98° for 30 sec, 50° for 30 sec, and 72° for 30 sec, followed by 72° for 7 min. Primers are summarized in Table S2. *ZYS3* promoter (*ZYS3p*^(−118 to +97)^ relative to TSS) was amplified with ZYS3p-*Xba*I-F and ZYS3UP-*Xho*I-REV. *ZYS3* promoter without ZYRE motifs (*ZYS3p*^(−94 to +97)^) was amplified with ZYS3-nomotif-F and ZYS3UP-*Xho*I-REV. *ZYS3* ZYRE motifs conjugated directly to 5′-UTR (*ZYS3p*^(−118 to −95: +1 to +95)^) were amplified with ZYS3-motif-5UTR-F and ZYS3UP-*Xho*I-REV. *ZYS3* 5′-UTR only (*ZYS3p*^(+1 to +97)^) was amplified with ZYS3-5UTR-F and ZYS3UP-*Xho*I-REV. *ZYS3* with a synthetic ZYRE sequence replacing its endogenous ZYREs (*Z-ZYS3p*^(−94 to +97)^) was amplified with ZYS3-Z-nomotif-F and ZYS3UP-*Xho*I-REV. The β-tubulin *TUB2* minimal promoter (*TUB2p*^(−53 to +113)^; upstream region containing the TATA box and 5′-UTR) was amplified with TM-*Xba*I-*Nde*I-F and TM-*Sal*I-R. A chimeric promoter of *ZYS3* ZYREs and *TUB2* minimal promoter (*ZYS3p*^(−118 to −95)^::*TUB2p*^(−53 to +113)^) was produced by PCR amplification with TM-*Xba*I-A-F and TM-*Sal*I-R. For *DMT4*, which has a ZYRE-like motif (CGTGACGTGAC) within its first intron, an in-frame translational fusion was made with its promoter, first exon first intron and a part of the second exon (*DMT4*) amplified with CMT1UP-*Xba*I-FWD and CMT1UP-*Xho*I-REV (Figure S1). The Ѱ-*DMT4*::*cgLuc* construct with mutated ZYRE was constructed by the one-step site-directed protocol (Zheng *et al.* 2004) from *DMT4*::*cgLuc* with DMT4-sub-ZYRE-F and DMT4-sub-ZYRE-R.

Resultant promoter-luciferase constructs were recombined with the plasmid pKS-aph7”-lox (containing a *loxP* site and the hygromycin resistant marker gene *aph7*”) via Cre/*lox*-mediated recombination (Ruecker *et al.* 2008) for the production of transformation vectors. Insert sequences of all plasmid samples for *C. reinhardtii* transformations were sequenced for validation using primers Cgluc-upXbaI or Glucrev4 (Table S2).

### Luciferase expression assay

Cells (CC-1690 21gr for *mt*+; CC-1691 6145c for *mt*−) were transformed using a modified version of a previously published electroporation protocol (Shimogawara *et al.* 1998). 300 ml cultures in TAP media were grown with bubbling aeration supplemented with 0.5% CO_2_ with continuous illumination from 150 µE red (625 nm) and 150 µE blue (465 nm) LED lights at 25° until they reached a density of 1 × 10^6^/ml. Cells were harvested by centrifugation at room temperature and resuspended at 3.5 × 10^8^ cells/ml in TAP with 50 mM sorbitol. Around 1 µg DNA and 1−5 × 10^7^ cells in 300 µl TAP with 50 mM sorbitol were placed into 4 mm cuvettes, prechilled on ice for 15 min, and then electroporated using a GenePulser Xcell (Bio-Rad Laboratories, Inc., Hercules, CA), with settings of 800 V, 25 µF, no resistance. Transformants were recovered in 10 ml TAP + 50 mM sorbitol in low light for 16 hr and then concentrated and plated on two TAP agar plates supplemented with 30 µg/ml hygromycin for selection. After 5 d single transformants were picked into 200 µl TAP media in clear 96-well plates, grown for 5 d in constant light, and then pinned onto TAP agar plates under 2 × 32 W fluorescent lights. After 5 d a toothpick full of cells from each pin spot was scraped from the plate and resuspended in 200 µl of nitrogen free-HSM (NF-HSM) to complete gametogenesis. A 50 µl aliquot of gametes from each transformant was mixed separately with testers of each mating-type (R3 *mt*+ or CJU10 *mt*−) and incubated under light for an additional 2 hr prior to measurement of luciferase activity. Mating activity, scored as agglutination between *plus* and *minus* gametes, was confirmed under the dissection microscope. Another 2 hr later, luminescence activity was measured as follows: Plates were processed and read in a FLUOstar Optima (BMG Labtech Gmbh, Offenberg, Germany) with automated injectors. Just prior to each reading, 100 µl of coelenterazine solution (10 µM coelenterazine, Gold Biotechnology; 100 mM Tris; pH 7.5; 500 mM NaCl; and 10 mM EDTA) was injected into a plate well containing 100 µl cell mixture, and luminescence was detected at the maximal gain setting (4095). Strains not expressing luciferase were used to measure background. Transformants that showed greater than fourfold more luminescence when mixed with gametes of the opposite mating-type testers *vs.* same mating-type testers were scored as positive for zygote-specific expression. In almost every case, when transformants were mixed with testers of the same mating-type the luminescence values were similar to background levels (Table S3).

### *Data* *availability*

The authors state that all data necessary for confirming the conclusions presented in the article are represented fully within the article.

## Results and Discussion

**Figure 1:**
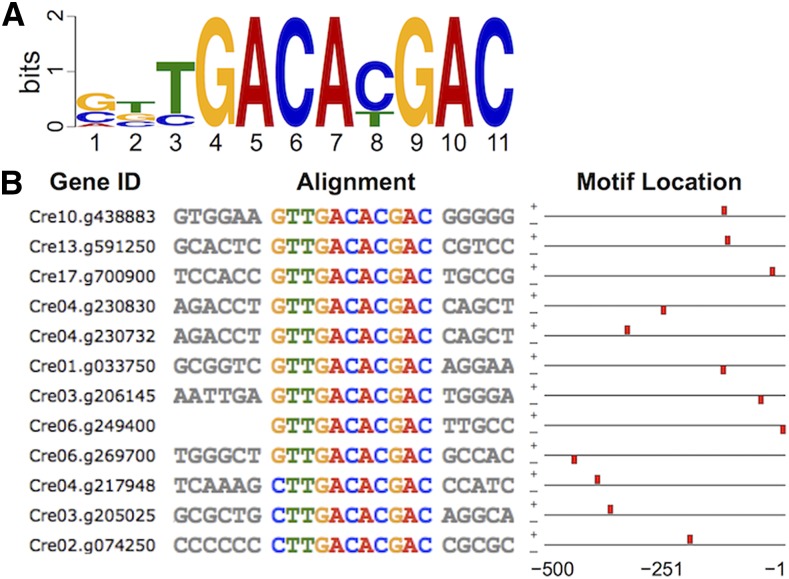
Identification of the ZYRE motif. (A) Logo representation of an enriched motif identified by MEME (Bailey and Elkan 1994) found upstream of start codons from a training set of the top 300 most highly expressed zygotic genes (Lopez *et al.* 2015). (B) Partial alignment of ZYRE motif-containing promoters in the training set. Block diagrams of the motif location and strand orientation (+ or −) relative to start codons with ZYREs represented by red boxes are shown.

**Figure 2:**
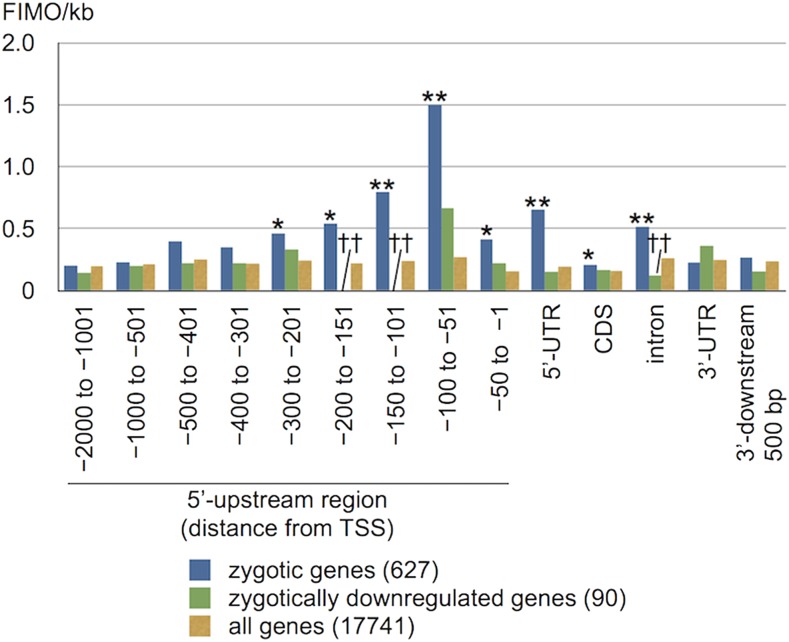
Relative frequencies of the ZYRE motif within different genic regions. Blue, green, or yellow bars stand for 627 zygotic genes, 90 zygotically downregulated genes, and all (17741) *C. reinhardtii* protein coding genes counted by FIMO (Grant *et al.* 2011). Single and double asterisks of zygotically upregulated genes indicate significant enrichment (*P* < 0.05 and < 0.01 respectively) in Student’s *t*-test compared to all genes; conversely, single and double daggers for zygotically downregulated genes, significant de-enrichment (*P* < 0.05 and < 0.01, respectively).

**Figure 3:**
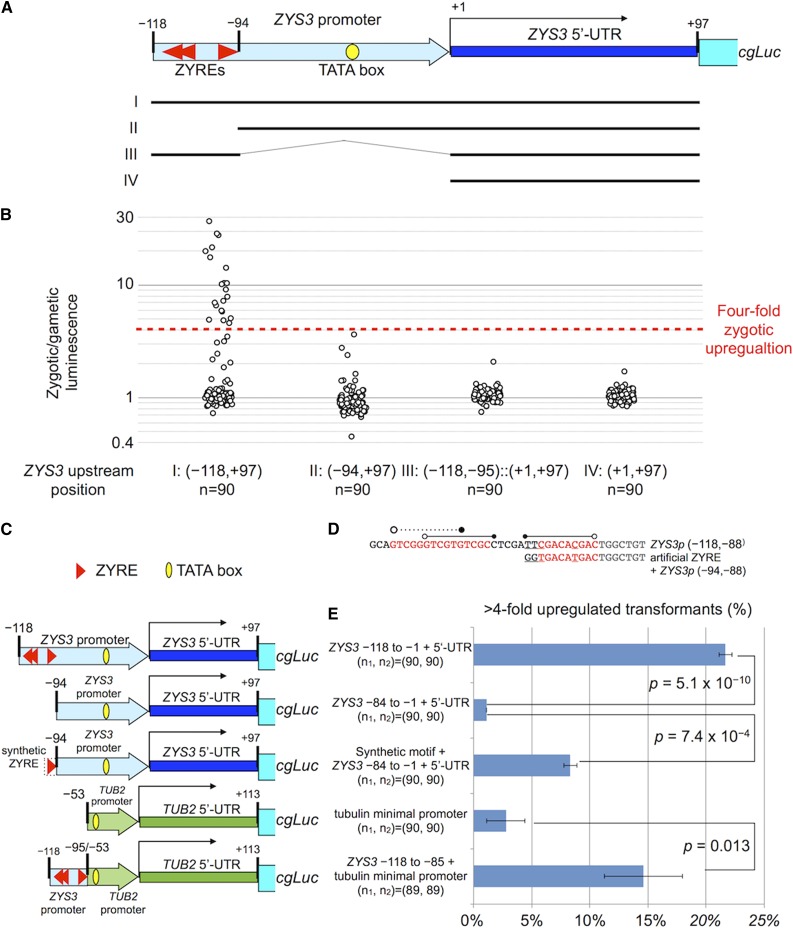
Functional characterization of the ZYRE motif using luciferase reporter constructs. (A) Schematic diagram of *ZYS3* promoter and deletion variants I−IV that were tested for zygotic luciferase expression. (B) Gametes from n = 90 *mt*+ transformants with indicated construct (I−IV) were mixed with *mt*+ gametes (negative control); or *mt*− gametes, allowed to mate, and then assessed for luciferase activity. Ratios of luminescence values of zygotic mixtures over negative controls are plotted. (C) Schematic diagram of promoters used to test sufficiency of ZYRE element for activating zygotic gene expression. Constructs were made using either the *ZYS3* promoter or a tubulin minimal promoter (Davies *et al.* 1992; Quinn and Merchant 1998) with the *ZYS3* ZYRE elements or a synthetic ZYRE element. (D) Sequences from *ZYS3* ZYRE elements and the synthetic ZYRE used in the constructs in panel C. (E) Percentage of transformants with zygotically upregulated luciferase expression (> fourfold compared to unmated control) from among ∼90 transformants tested in each of two biological replicates. The *P*-values for χ^2^ tests between ZYRE and non-ZYRE control constructs are indicated. Analysis of variance with Tukey’s test (95% confidence level) was also performed for the first three constructs: between *ZYS3p*^(−118 to +97)^::*cgLuc* and *ZYS3p*^(−94 to +97)^::*cgLuc*, *P* = 5.7 × 10^−5^; between synthetic ZYRE+*ZYS3p*^(−94 to +97)^::*cgLuc* and *ZYS3p*^(−94 to +97)^::*cgLuc*, *P* = 0.013; between *ZYS3p*^(−118 to +97)^::*cgLuc* and synthetic ZYRE+*ZYS3p*^(−94 to +97)^::*cgLuc*, *P* = 2.8 × 10^−4^.

**Figure 4:**
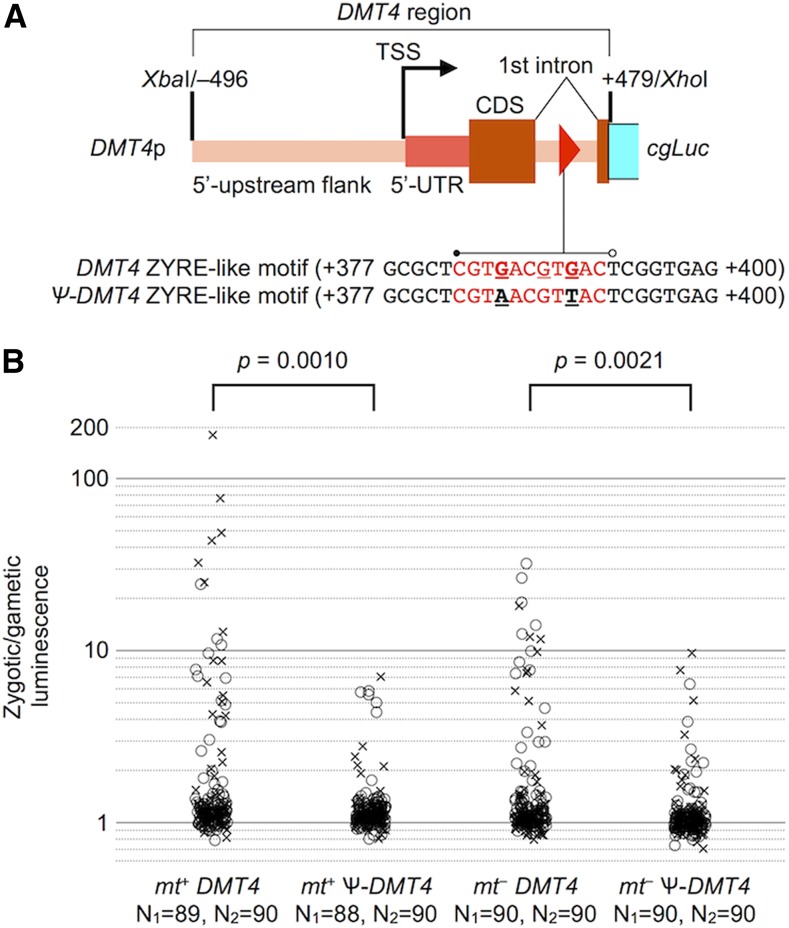
Reporter gene assay for *DMT4* intronic ZYRE motif. (A) Diagram of reporter construct used for native (*DMT4p*) and mutated (Ѱ*-DMT4p*) *DMT4* including the promoter region (496 bp), first exon, first intron containing a ZYRE-like motif (red triangle), and short extension of the second exon. The native and mutated versions of the DMT4 ZYRE motif are shown below. (B) Plot showing fold-changes of zygotic over gametic luminescence for two sets of biological replicates in each of two different parental strains (*mt+* or *mt−*) graphed as in Figure 3. Circle and cross symbols show data points for the first (N_1_) and second (N_2_) sets of transformants respectively. *P*-values for pooled replicate data (χ^2^ test) are indicated above each set of experiments.

We compiled 500 bp regions upstream of start codons for the 300 most highly induced zygotic genes ranked based on the ratio of zygotic expression to the maximum expression among all nonzygotic samples (Table S1; Lopez *et al.* 2015). MEME (Bailey and Elkan 1994) was used to identify enriched motifs within the 300 zygotic promoters (Table S4). Out of ten significant MEME motifs eight were present in a small number of genes, but two reached the count limit of 50. One of these abundant motifs was a low-complexity G-rich sequence that is widespread in the *C*. *reinhardtii* genome and was not pursued further. The other high-frequency motif was an 11-mer with the sequence complexity characteristic of a *bona fidecis*-regulatory element, YGACAYGAC, that we named ZYRE (Figure 1) and was subsequently found within 485/627 (77%) early zygotic genes (Table S1). ZYRE is similar to but more refined and statistically supported than the CGtGACATGaCC motif previously identified from a limited set of zygotic genes (Lee *et al.* 2008). Occurrence and distribution of ZYRE was enumerated by FIMO (Grant *et al.* 2011) across the whole genome and its enrichment in zygotic genes was assessed by location within flanking regions, 5′- and 3′-UTRs, CDSs, and introns (Table S1 and Figure 2). The strongest enrichment of ZYRE was found upstream of zygotic genes with a peak in the –51 to –100 promoter regions relative to the TSS, a location that is consistent with possible function as a transcription factor binding site and *cis*-regulatory element (Figure 2). In addition significant enrichment of ZYRE was also found in 5′-UTRs and introns (Figure 2). Indeed, as described below, a reporter construct made with an intronic ZYRE element from the zygotic gene *DMT4* (Lopez *et al.* 2015) was able to confer zygotic expression suggesting that the element can work within transcribed regions and not just in promoters (Figure 3 and Figure 4). Conversely, 90 genes that are suppressed in zygotes (genes expressed in vegetative or gametic cells but silenced in zygotes; Table S1) show significant de-enrichment of ZYRE in their promoters (–300 to –201: *P* = 1.1 × 10^−43^; –200 to –101: *P* = 1.2 × 10^−46^) and introns (*P* = 1.9 × 10^−5^; Figure 2). These observations support the idea that ZYREs in promoter and noncoding genic regions promote zygotic gene expression. No strand orientation bias was observed for ZYRE, a result that is also consistent with it acting as a transcriptional enhancer (Table 1). Some genes that are not specifically expressed in zygotes also have ZYREs (Table 1). As is the case with other *cis*-regulatory motifs, only a fraction of genes proximal to them in the genome display the expression pattern associated with that motif (Beer and Tavazoie 2004; Hughes and de Boer 2013). We also note that our search would not have identified genes that are controlled by ZYRE as well as additional elements that promote expression at other life cycle stages.

**Table 1:** Comparison of ZYRE occurrence over the *C. reinhardtii* genome

	**FIMO Count**	**Sequence (bp)**	**FIMO/Sequence kb**	**FIMO/Gene**	**+/– Strand Ratio**
Whole genome	17,522	84,725,637	0.207	—	8880/8642
500 bp upstream of zygotic 627 CDS	176	313,500	0.561	0.230	87/89
500 bp upstream of All (17741) CDS	1955	8,865,235	0.221	0.102	963/992

**Figure 5:**
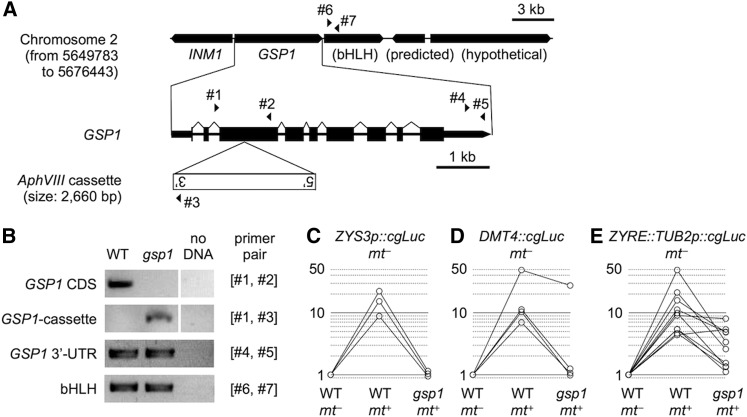
ZYRE-dependent zygotic transcription requires *GSP1*. (A) Schematic diagram of *GSP1* locus and surrounding regions. The *AphVIII* insertion cassette in exon 3 of *GSP1* in strain LMJ.SG0182.003395 is shown below. Triangles with numbers indicate positions of primers used for PCR amplification in panel B. (B) PCR genotyping of *gsp1* mutant locus. Images for “no DNA” came from the same gel with intervening lanes cropped. (C−E) Three sets of *mt*− transformants that were positive for zygotic upregulation were mixed with negative controls (mixed with wild-type (WT) *mt−* gametes), positive controls with WT *mt*+ gametes, and the *gsp1* mutant *mt*+ gametes. Numbers of transformants are as follows: (C) *ZYS3p*::*cgLuc*: N = 3; (D) *DMT4*::*cgLuc*: N = 4; (E) *ZYRE*::*TUB2p*::*cgLuc*: N = 10.

To test whether the ZYRE motif is necessary for zygotic expression *in vivo*, we employed a luciferase reporter assay with promoter sequences driving expression of a codon-optimized *Gaussia princeps* luciferase gene (*cgLuc*; Ruecker *et al.* 2008). We made luciferase reporter constructs for two ZYRE-containing zygotic genes, *ZYS3* (Kuriyama *et al.* 1999) and *DMT4* (Lopez *et al.* 2015). *ZYS3* contains three ZYRE motifs in a 25 bp region from –118 to –94 relative to its TSS (Figure 3A). *DMT4* (encoding a putative chloroplast-targeted DNA methyltransferase; Lopez *et al.* 2015) has a ZYRE-like motif (CGTGACGTGAC; one base mismatch from Figure 1, hereafter treated as ZYRE) in its first intron (Figure 4A). These constructs and a control construct containing the *ZYS3* promoter without ZYRE motifs were each transformed into a *mt+* strain and randomly chosen colonies were grown and induced to become gametes. The transformed gametes were mixed with equal numbers of either *mt+* or *mt−* mating test strains and shortly thereafter newly formed zygotes were assessed for luciferase activity (see *Materials and Methods*, Figure 3B, Figure 4, and Table S3). For both ZYRE-containing reporters we observed a fraction of transformants (∼10%) that had zygotic upregulation of luciferase, while the majority of remaining transformants showed either low or background levels of luciferase activity. The observed fraction of positive transformants in our experiments is consistent with results from other studies where random integration of transgenes often results in silencing (Cerutti *et al.* 1997; Fuhrmann *et al.* 1999; Figure 3B, construct I, Figure 4, and Table S3). Importantly, upregulation of zygotic luciferase activity was rarely observed in transformants that had missing or mutated ZYRE elements (Figure 3B, Figure 4B, construct II, and Table S3). Thus, ZYRE elements can function as zygotic transcriptional enhancers both upstream of and within transcription units. Similar results as those described above were obtained when reporters were tested in *mt*− strains meaning that the parental gamete type in which the ZYRE reporter resides does not influence its activity (Figure 4B and Figure 5, C−E).

We next examined the *ZYS3* proximal promoter region and 5′ UTR (−118 to +97) in more detail. The *ZYS3* promoter contains three ZYRE motifs in a 25 bp region from –94 to –118 (Figure 3A, construct I, and Figure 3, C and E). When the 25 bp ZYRE-containing region was deleted zygotic expression was lost (Figure 3A, construct II, and Figure 3, C and E). However, when replaced with a synthetic ZYRE sequence zygotic expression was restored (Figure 3, C and E). The construct with the original 25 bp region contains three ZYREs (one in forward and two in reverse orientation) and gave more transformants with zygotic luminescence than the single synthetic ZYRE suggesting that multiple ZYREs may have an additive or synergistic effect on expression. When the more proximal region of the promoter was deleted zygotic expression was also lost, possibly due to the elimination of core promoter elements such as the TATA box (Figure 3A, construct III) or because this region has additional *cis*-regulatory motifs that also contribute to zygotic expression. Together these data show that ZYRE motifs in the *ZYS3* promoter are necessary to confer zygotic upregulation.

To determine whether ZYRE motifs are sufficient to drive zygotic expression we fused the 25 bp ZYRE motifs from *ZYS3* (–118 to –94) to a tubulin minimal promoter that has been employed previously in reporter constructs (Davies *et al.* 1992; Davies and Grossman 1994; Quinn and Merchant 1998). Transformants with the *cgLuc* gene fused only to the tubulin basal promoter did not show any gametic or zygotic expression above background. However, addition of the 25 bp region containing ZYRE motifs from *ZYS3* conferred zygotic expression to this construct leading us to conclude that when coupled with basal promoter elements the ZYRE motif is sufficient to drive zygotic gene expression (Figure 3, C and E). Our results also suggest that the putative cAMP responsive element (typically “TGACGTCA”) that was previously found upstream of *ZYS3* at –700 from the TSS and is missing in our constructs (Uchida *et al.* 2004) is not essential for zygotic regulation, though it might have redundant or other roles that remain to be determined.

A candidate for a transcription factor that recognizes ZYRE is the heterodimeric GSP1-GSM1 homeodomain protein whose subunits are expressed separately in *plus* (*GSP1*) and *minus* (*GSM1*) gametes respectively, and which heterodimerize and enter the nucleus upon fertilization (Kurvari *et al.* 1998; Zhao *et al.* 2001; Lee *et al.* 2008; Nishimura *et al.* 2012). To test whether ZYRE-regulated transcription requires GSP1-GSM1, we compared the zygotic luminescence of *mt*− transformants containing one of three different ZYRE-dependent reporters when fertilized by either wild-type *mt+* gametes or by *gsp1 mt*+ gametes that contain an insertional mutation in the third exon of the *GSP1* gene (Figure 5, A and B). Fertilization rates using wild-type *mt+* gametes were > 50% while with *gsp1 mt+* gametes they were > 70%, yet zygotic induction of reporter gene transcription when *gsp1 mt+* gametes were used for mating was absent or lower than in matings to the wild-type *mt+* parent in 14/17 transformants (Figure 5, C−E). We note that in most of our experiments a small fraction of reporter construct transformants acquired a mating-dependent expression pattern even when they only had a minimal promoter with no ZYRE motif or other enhancers (Figure 3E), most likely due to integration near genes that are upregulated during mating. We attribute the three mating-dependent but GSP1-independent reporter strains we observed in Figure 5, D and E to such integration events that occurred at a similar frequency using enhancerless constructs (Figure 3E). Taken together our results show that ZYRE-controlled zygotic gene expression depends on GSP1 under the direct or indirect control of GSP1-GSM1 heterodimers.

**Figure 6:**
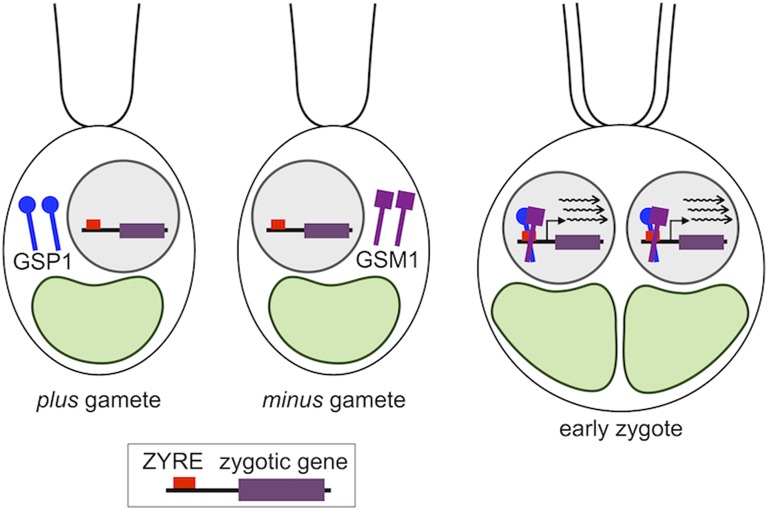
Minimalist model for ZYRE motif as a direct binding site for the zygotic heterodimeric transcription factor GSP1-GSM1 whose subunits are expressed in a mating-type specific pattern. Upon fertilization GSP1-GSM1 heterodimerize, and translocate to the nucleus where they can bind to ZYRE-containing promoters and activate gene expression.

The simplest scenario for regulation of early zygotic gene expression would involve direct binding of GSP1-GSM1 heterodimers to ZYREs as a transcriptional activator (Figure 6), an idea that could be directly tested through *in vitro* or *in vivo* binding studies. Homologs of GSM1 and GSP1 in the KNOX and BELL subfamilies in land plants are known to heterodimerize and recognize motifs whose half-sites are composed of eight base pairs (Hake *et al.* 2004). The ZYRE motif that we characterized here shares a YGAC submotif with the previously identified TGAC submotif present in the binding sites for plant KNOX-BELL proteins. However, GSP1 differs in a critical DNA binding region in the third helix of its homeodomain with a WFTN motif instead of WFIN or WFVN that is found in other KNOX-BELL proteins including GSM1 (Lee *et al.* 2008). The DNA binding sites of GSP1-GSM1 and the peptide sequences that contribute to its specificity remain to be determined, but if GSP1-GSM1 does prove to bind to ZYRE motifs the study of this interaction might contribute to a deeper understanding of what determines homeodomain-DNA binding specificity.

Many (77%) but not all *C. reinhardtii* early zygotic genes contain ZYRE motifs (Table S1). It is possible that the motif search criteria we used missed partially degenerate ZYRE-like sequences in these genes, as was the case with *DMT4*, or that alternative GSP1-GSM1 binding sites exist for those genes. In addition some zygote-specific genes may be regulated indirectly by zygotic transcription factors that act downstream of or independent of GSP1-GSM1. Among our zygote-specific genes (Table S1), there are five that encode putative transcription factors: *ZYS1A* and *ZYS1B* (Uchida *et al.* 1993, 1999, 2004), *RLS7* (Duncan *et al.* 2007), *EZY18* (Kubo *et al.* 2008), and a σ^70^-like putative transcription factor (Cre03.g193400). An interesting area of future study will be to determine whether motifs that are present in zygotic promoters (Table S4) which do not contain detectable ZYREs are recognized by GSP1 and GSM1 or other zygotic transcription factors.

## 

## Supplementary Material

Supplemental Material
