# Supplementary material for: Identification and Characterization of a cis-Regulatory Element for Zygotic Gene Expression in Chlamydomonas reinhardtii
Source: G3 (Bethesda). 2016 Mar 23;6(6):1541–8. doi: 10.1534/g3.116.029181 (PMC4889651; doi:10.1534/g3.116.029181)
Supplement: Supplemental Material [file supp_6_6_1541__index.html]

Supplemental Material 

# Identification and Characterization of a *cis*-Regulatory Element for Zygotic Gene Expression in *Chlamydomonas reinhardtii*

## Supplemental Material for Hamaji *et al.*, 2016

**Files in this Data Supplement:**

- Figure S1 - Sequences of transgenes used in the luciferase expression experiments. (.pdf, 127 KB)
- Table S2 - Primers used in this study. (.pdf, 61 KB)
- Table S4 - MEME results. (.pdf, 210 KB)
- Table S1 - Zygotically upregulated (Sheet2) and downregulated (Sheet3) genes identified from transcriptome analysis (Lopez et al. 2015). (.xlsx, 108 KB)
- Table S3 - Individual numerical data for promoter-luciferase assays. (.xlsx, 92 KB)
