# Supplementary material for: Identification and Characterization of a cis-Regulatory Element for Zygotic Gene Expression in Chlamydomonas reinhardtii
Source: G3 (Bethesda). 2016 Mar 23;6(6):1541–8. doi: 10.1534/g3.116.029181 (PMC4889651; doi:10.1534/g3.116.029181)
Supplement: Supplemental Material [file supp_g3.116.029181_FigureS1.pdf]

ZYS3p$^{(-118\text{ to }+97)}$*::cgLuc*; Construct I in Figure 3A, 3B

```
TctagaGCAGTCGGGTCGTGTCGCCTCGATTCGACACGACTGGCTGTAGC   < 50
GCCCGGCCCGCATGGGGCATGCCCGGCGCCCTATAAAACGCGAGCGGAGT   < 100
GCCGAACCCTGCATTCACACAGACCTTCTTCCTCTCGCTTACCTTGTGAA   < 150
GCACTGATAGCCGGGCATCCGCCTCTCCCTTCCCCTCGGGCTGCGCGCCT   < 200
CTCCACCGAACACATCCCTCGctcgaGATGGGCGTGAAGGTGCTGTTCGC   < 250
CCTGATCTGCATCGCCGTCGCCGAGGCGAAGCCGACGGAGAACAACGAGG   < 300
ACTTCAACATTGTCGCCGTGGCGTCCAACTTTGCCACCACCGATCTGGAC   < 350
GCCGACCGCGGCAAGCTGCCGGGCAAGAAGCTGCCCCTGGAGGTCCTCAA   < 400
GGAAATGGAGGCGAACGCCCGGAAGGCCGGCTGCACCCGCGGCTGCCTAA   < 450
TCTGCCTGTCCCACATCAAGTGCACGCCGAAGATGAAGAAGTTCATTCCC   < 500
GGCCGGTGCCACACCTACGAGGGCGACAAGGAGTCCGCGCAGGGCGGCAT   < 550
TGGCGAGGCGATCGTGGACATCCCCGAGATTCCGGGCTTCAAGGACCTGG   < 600
AGCCGATGGAGCAGTTCATCGCCCAGGTGGACCTGTGCGTGGACTGCACG   < 650
ACCGGCTGTCTGAAGGGCCTGGCCAATGTGCAGTGTTCCGACCTGCTCAA   < 700
GAAGTGGCTGCCCCAGCGCTGTGCCACCTTCGCCAGCAAGATCCAGGGCC   < 750
AGGTGGACAAGATTAAGGGCGCTGGCGGCGATACGTAAGGATCCCCGCTC   < 800
CGTGTAAATGGAGGCGCTCGTTGATCTGAGCCTTGCCCCCTGACGAACGG   < 850
CGGTGGATGGAAGATACTGCTCTCAAGTGCTGAAGCGGTAGCTTAGCTCC   < 900
CCGTTTCGTGCTGATCAGTCTTTTTCAACACGTAAAAAGCGGAGGAGTTT   < 950
TGCAATTTTGTTGGTTGTAACGATCCTCCGTTGATTTTGGCCTCTTTCTC   < 1000
CATGG                                                < 1005
```

```
Features :
ZYS3 region       : [6 : 222]
ZYS3 5'-UTR       : [125 : 221]
zygotic motif     : [32 : 40]
zygotic motif     : [23 : 15]
TATA box          : [82 : 87]
cgLuc CDS         : [228 : 788]
3-UTR             : [784 : 1000]
```

ZYS3p$^{(-94\text{ to }+97)}$ *::cgLuc*; Construct II in Figure 3A, 3B

```
TctagaTGGCTGTAGCGCCCGGCCCGCATGGGGCATGCCCGGCGCCCTAT   < 50
AAAACGCGAGCGGAGTGCCGAACCCTGCATTCACACAGACCTTCTTCCTC   < 100
TCGCTTACCTTGTGAAGCACTGATAGCCGGGCATCCGCCTCTCCCTTCCC   < 150
CTCGGGCTGCGCGCCTCTCCACCGAACACATCCCTCGctcgaGATGGGCG   < 200
TGAAGGTGCTGTTCGCCCTGATCTGCATCGCCGTCGCCGAGGCGAAGCCG   < 250
ACGGAGAACAACGAGGACTTCAACATTGTCGCCGTGGCGTCCAACTTTGC   < 300
CACCACCGATCTGGACGCCGACCGCGGCAAGCTGCCGGGCAAGAAGCTGC   < 350
CCCTGGAGGTCCTCAAGGAAATGGAGGCGAACGCCCGGAAGGCCGGCTGC   < 400
ACCCGCGGCTGCCTAATCTGCCTGTCCCACATCAAGTGCACGCCGAAGAT   < 450
GAAGAAGTTCATTCCCGGCCGGTGCCACACCTACGAGGGCGACAAGGAGT   < 500
CCGCGCAGGGCGGCATTGGCGAGGCGATCGTGGACATCCCCGAGATTCCG   < 550
GGCTTCAAGGACCTGGAGCCGATGGAGCAGTTCATCGCCCAGGTGGACCT   < 600
GTGCGTGGACTGCACGACCGGCTGTCTGAAGGGCCTGGCCAATGTGCAGT   < 650
GTTCCGACCTGCTCAAGAAGTGGCTGCCCCAGCGCTGTGCCACCTTCGCC   < 700
AGCAAGATCCAGGGCCAGGTGGACAAGATTAAGGGCGCTGGCGGCGATAC   < 750
GTAAGGATCCCCGCTCCGTGTAAATGGAGGCGCTCGTTGATCTGAGCCTT   < 800
GCCCCCTGACGAACGGCGGTGGATGGAAGATACTGCTCTCAAGTGCTGAA   < 850
GCGGTAGCTTAGCTCCCCGTTTCGTGCTGATCAGTCTTTTTCAACACGTA   < 900
AAAAGCGGAGGAGTTTTGCAATTTTGTTGGTTGTAACGATCCTCCGTTGA   < 950
TTTTGGCCTCTTTCTCCATGG                                < 971
```

```
Features :
ZYS3 region    : [6 : 188]
TATA box       : [48 : 53]
ZYS3 5'-UTR    : [91 : 187]
cgLuc CDS      : [194 : 754]
3-UTR          : [750 : 966]
```

ZYS3p$^{(-118 \text{ to } -95\text{: } +1 \text{ to } +95)}$*::cgLuc*; Construct III in Figure 3A, 3B

```
TctagaGCAGTCGGGTCGTGTCGCCTCGATTCGACACGACCTTCTTCCTC  < 50
TCGCTTACCTTGTGAAGCACTGATAGCCGGGCATCCGCCTCTCCCTTCCC  < 100
CTCGGGCTGCGCGCCTCTCCACCGAACACATCCCTCGctcgaGATGGGCG  < 150
TGAAGGTGCTGTTCGCCCTGATCTGCATCGCCGTCGCCGAGGCGAAGCCG  < 200
ACGGAGAACAACGAGGACTTCAACATTGTCGCCGTGGCGTCCAACTTTGC  < 250
CACCACCGATCTGGACGCCGACCGCGGCAAGCTGCCGGGCAAGAAGCTGC  < 300
CCCTGGAGGTCCTCAAGGAAATGGAGGCGAACGCCCGGAAGGCCGGCTGC  < 350
ACCCGCGGCTGCCTAATCTGCCTGTCCCACATCAAGTGCACGCCGAAGAT  < 400
GAAGAAGTTCATTCCCGGCCGGTGCCACACCTACGAGGGCGACAAGGAGT  < 450
CCGCGCAGGGCGGCATTGGCGAGGCGATCGTGGACATCCCCGAGATTCCG  < 500
GGCTTCAAGGACCTGGAGCCGATGGAGCAGTTCATCGCCCAGGTGGACCT  < 550
GTGCGTGGACTGCACGACCGGCTGTCTGAAGGGCCTGGCCAATGTGCAGT  < 600
GTTCCGACCTGCTCAAGAAGTGGCTGCCCCAGCGCTGTGCCACCTTCGCC  < 650
AGCAAGATCCAGGGCCAGGTGGACAAGATTAAGGGCGCTGGCGGCGATAC  < 700
GTAAGGATCCCCGCTCCGTGTAAATGGAGGCGCTCGTTGATCTGAGCCTT  < 750
GCCCCCTGACGAACGGCGGTGGATGGAAGATACTGCTCTCAAGTGCTGAA  < 800
GCGGTAGCTTAGCTCCCCGTTTCGTGCTGATCAGTCTTTTTCAACACGTA  < 850
AAAAGCGGAGGAGTTTTGCAATTTTGTTGGTTGTAACGATCCTCCGTTGA  < 900
TTTTGGCCTCTTTCTCCATGG                               < 921
```

```
Features :
ZYS3 region     : [6 : 138]
ZYS3 5'-UTR     : [41 : 137]
zygotic motif   : [32 : 40]
zygotic motif   : [18 : 10]
zygotic motif   : [23 : 15]
cgLuc CDS       : [144 : 704]
3-UTR           : [700 : 916]
```

ZYS3p$^{(+1 \text{ to } +97)}$ *::cgLuc*; Construct IV in Figure 3A, 3B

```
TctagaaCTTCTTCCTCTCGCTTACCTTGTGAAGCACTGATAGCCGGGCA   < 50
TCCGCCTCTCCCTTCCCCTCGGGCTGCGCGCCTCTCCACCGAACACATCC   < 100
CTCGctcgaGATGGGCGTGAAGGTGCTGTTCGCCCTGATCTGCATCGCCG   < 150
TCGCCGAGGCGAAGCCGACGGAGAACAACGAGGACTTCAACATTGTCGCC   < 200
GTGGCGTCCAACTTTGCCACCACCGATCTGGACGCCGACCGCGGCAAGCT   < 250
GCCGGGCAAGAAGCTGCCCCTGGAGGTCCTCAAGGAAATGGAGGCGAACG   < 300
CCCGGAAGGCCGGCTGCACCCGCGGCTGCCTAATCTGCCTGTCCCACATC   < 350
AAGTGCACGCCGAAGATGAAGAAGTTCATTCCCGGCCGGTGCCACACCTA   < 400
CGAGGGCGACAAGGAGTCCGCGCAGGGCGGCATTGGCGAGGCGATCGTGG   < 450
ACATCCCCGAGATTCCGGGCTTCAAGGACCTGGAGCCGATGGAGCAGTTC   < 500
ATCGCCCAGGTGGACCTGTGCGTGGACTGCACGACCGGCTGTCTGAAGGG   < 550
CCTGGCCAATGTGCAGTGTTCCGACCTGCTCAAGAAGTGGCTGCCCCAGC   < 600
GCTGTGCCACCTTCGCCAGCAAGATCCAGGGCCAGGTGGACAAGATTAAG   < 650
GGCGCTGGCGGCGATACGTAAGGATCCCCGCTCCGTGTAAATGGAGGCGC   < 700
TCGTTGATCTGAGCCTTGCCCCCTGACGAACGGCGGTGGATGGAAGATAC   < 750
TGCTCTCAAGTGCTGAAGCGGTAGCTTAGCTCCCCGTTTCGTGCTGATCA   < 800
GTCTTTTTCAACACGTAAAAAGCGGAGGAGTTTTGCAATTTTGTTGGTTG   < 850
TAACGATCCTCCGTTGATTTTGGCCTCTTTCTCCATGG               < 888
```

```
Features :
ZYS3 5'-UTR    : [8 : 104]
cgLuc CDS      : [111 : 671]
3-UTR          : [667 : 883]
```

Z-ZYS3p$^{(-94\ \mathrm{to}\ +97)}$ *::cgLuc*; synthetic ZYRE substitution to *ZYS3* promoter in Figure 3C, 3D, 3E

```
TctagaGGTGACATGACTGGCTGTAGCGCCCGGCCCGCATGGGGCATGCC   < 50
CGGCGCCCTATAAAACGCGAGCGGAGTGCCGAACCCTGCATTCACACAGA   < 100
CCTTCTTCCTCTCGCTTACCTTGTGAAGCACTGATAGCCGGGCATCCGCC   < 150
TCTCCCTTCCCCTCGGGCTGCGCGCCTCTCCACCGAACACATCCCTCGct   < 200
cgaGATGGGCGTGAAGGTGCTGTTCGCCCTGATCTGCATCGCCGTCGCCG   < 250
AGGCGAAGCCGACGGAGAACAACGAGGACTTCAACATTGTCGCCGTGGCG   < 300
TCCAACTTTGCCACCACCGATCTGGACGCCGACCGCGGCAAGCTGCCGGG   < 350
CAAGAAGCTGCCCCTGGAGGTCCTCAAGGAAATGGAGGCGAACGCCCGGA   < 400
AGGCCGGCTGCACCCGCGGCTGCCTAATCTGCCTGTCCCACATCAAGTGC   < 450
ACGCCGAAGATGAAGAAGTTCATTCCCGGCCGGTGCCACACCTACGAGGG   < 500
CGACAAGGAGTCCGCGCAGGGCGGCATTGGCGAGGCGATCGTGGACATCC   < 550
CCGAGATTCCGGGCTTCAAGGACCTGGAGCCGATGGAGCAGTTCATCGCC   < 600
CAGGTGGACCTGTGCGTGGACTGCACGACCGGCTGTCTGAAGGGCCTGGC   < 650
CAATGTGCAGTGTTCCGACCTGCTCAAGAAGTGGCTGCCCCAGCGCTGTG   < 700
CCACCTTCGCCAGCAAGATCCAGGGCCAGGTGGACAAGATTAAGGGCGCT   < 750
GGCGGCGATACGTAAGGATCCCCGCTCCGTGTAAATGGAGGCGCTCGTTG   < 800
ATCTGAGCCTTGCCCCCTGACGAACGGCGGTGGATGGAAGATACTGCTCT   < 850
CAAGTGCTGAAGCGGTAGCTTAGCTCCCCGTTTCGTGCTGATCAGTCTTT   < 900
TTCAACACGTAAAAAGCGGAGGAGTTTTGCAATTTTGTTGGTTGTAACGA   < 950
TCCTCCGTTGATTTTGGCCTCTTTCTCCATGG                     < 982
```

```
Features :
zygotic motif    : [9 : 17]
ZYS3 region      : [18 : 199]
ZYS3 5'-UTR      : [102 : 198]
cgLuc CDS        : [205 : 765]
3-UTR            : [761 : 977]
```

TUB2p$^{(-53\text{ to }+113)}$ *::cgLuc*; tubulin minimal promoter in Figure 3C, 3E

```
>Tubulin minimal promoter
TctagacatatgCTGTTTAAATAGCCAGGCCCCGATTGCAAAGACATTA   < 50
TAGCGAGCTACCAAAGCCATATTCAAACACCTAGATCACTACCACTTCTA  < 100
CACAGGCCACTCGAGCTTGTGATCGCACTCCGCTAAGGGGGCGCCTCTTC  < 150
CTCTTCGTTTCAGTCACAACCCGCAAACgtcgaGATGGGCGTGAAGGTGC  < 200
TGTTCGCCCTGATCTGCATCGCCGTCGCCGAGGCGAAGCCGACGGAGAAC  < 250
AACGAGGACTTCAACATTGTCGCCGTGGCGTCCAACTTTGCCACCACCGA  < 300
TCTGGACGCCGACCGCGGCAAGCTGCCGGGCAAGAAGCTGCCCCTGGAGG  < 350
TCCTCAAGGAAATGGAGGCGAACGCCCGGAAGGCCGGCTGCACCCGCGGC  < 400
TGCCTAATCTGCCTGTCCCACATCAAGTGCACGCCGAAGATGAAGAAGTT  < 450
CATTCCCGGCCGGTGCCACACCTACGAGGGCGACAAGGAGTCCGCGCAGG  < 500
GCGGCATTGGCGAGGCGATCGTGGACATCCCCGAGATTCCGGGCTTCAAG  < 550
GACCTGGAGCCGATGGAGCAGTTCATCGCCCAGGTGGACCTGTGCGTGGA  < 600
CTGCACGACCGGCTGTCTGAAGGGCCTGGCCAATGTGCAGTGTTCCGACC  < 650
TGCTCAAGAAGTGGCTGCCCCAGCGCTGTGCCACCTTCGCCAGCAAGATC  < 700
CAGGGCCAGGTGGACAAGATTAAGGGCGCTGGCGGCGATACGTAAGGATC  < 750
CCCGCTCCGTGTAAATGGAGGCGCTCGTTGATCTGAGCCTTGCCCCCTGA  < 800
CGAACGGCGGTGGATGGAAGATACTGCTCTCAAGTGCTGAAGCGGTAGCT  < 850
TAGCTCCCCGTTTCGTGCTGATCAGTCTTTTTCAACACGTAAAAAGCGGA  < 900
GGAGTTTTGCAATTTTGTTGGTTGTAACGATCCTCCGTTGATTTTGGCCT  < 950
CTTTCTCCATGG                                        < 962
```

```
Features :
TUB2 region    : [6 : 179]
TATA box       : [16 : 21]
TUB2 5'UTR     : [66 : 178]
cgLUC          : [185 : 742]
3-UTR          : [741 : 957]
```

ZYS3p$^{(-118 \text{ to } -95)}$ *::TUB2p*$^{(-53 \text{ to } +113)}$*::cgLuc*; chimeric promoter made from *ZYS3* and *TUB2* tubulin minimal promoter in Figure 3C, 3E, 5E

```
TctagaGCAGTCGGGTCGTGTCGCCTCGATTCGACACGACCTGTTTAAAT  < 50
AGCCAGGCCCCCGATTGCAAAGACATTATAGCGAGCTACCAAAGCCATAT  < 100
TCAAACACCTAGATCACTACCACTTCTACACAGGCCACTCGAGCTTGTGA  < 150
TCGCACTCCGCTAAGGGGGCGCCTCTTCCTCTTCGTTTCAGTCACAACCC  < 200
GCAAACgtcgaGATGGGCGTGAAGGTGCTGTTCGCCCTGATCTGCATCGC  < 250
CGTCGCCGAGGCGAAGCCGACGGAGAACAACGAGGACTTCAACATTGTCG  < 300
CCGTGGCGTCCAACTTTGCCACCACCGATCTGGACGCCGACCGCGGCAAG  < 350
CTGCCGGGCAAGAAGCTGCCCCTGGAGGTCCTCAAGGAAATGGAGGCGAA  < 400
CGCCCGGAAGGCCGGCTGCACCCGCGGCTGCCTAATCTGCCTGTCCCACA  < 450
TCAAGTGCACGCCGAAGATGAAGAAGTTCATTCCCGGCCGGTGCCACACC  < 500
TACGAGGGCGACAAGGAGTCCGCGCAGGGCGGCATTGGCGAGGCGATCGT  < 550
GGACATCCCCGAGATTCCGGGCTTCAAGGACCTGGAGCCGATGGAGCAGT  < 600
TCATCGCCCAGGTGGACCTGTGCGTGGACTGCACGACCGGCTGTCTGAAG  < 650
GGCCTGGCCAATGTGCAGTGTTCCGACCTGCTCAAGAAGTGGCTGCCCCA  < 700
GCGCTGTGCCACCTTCGCCAGCAAGATCCAGGGCCAGGTGGACAAGATTA  < 750
AGGGCGCTGGCGGCGATACGTAAGGATCCCCGCTCCGTGTAAATGGAGGC  < 800
GCTCGTTGATCTGAGCCTTGCCCCCTGACGAACGGCGGTGGATGGAAGAT  < 850
ACTGCTCTCAAGTGCTGAAGCGGTAGCTTAGCTCCCCGTTTCGTGCTGAT  < 900
CAGTCTTTTTCAACACGTAAAAAGCGGAGGAGTTTTGCAATTTTGTTGGT  < 950
TGTAACGATCCTCCGTTGATTTTGGCCTCTTTCTCCATGG            < 990
```

```
Features :
ZYS3 region      : [6 : 40]
zygotic motif    : [18 : 10]
zygotic motif    : [23 : 15]
zygotic motif    : [32 : 40]
TUB2 region      : [41 : 207]
TUB2 5'UTR       : [94 : 206]
TATA box         : [44 : 49]
cgLuc CDS        : [213 : 773]
3-UTR            : [769 : 985]
```

DMT4$^{(-498 \text{ to } +479)}$ *::cgLuc*; **Error! Reference source not found.**

```
TctagaGTGAGCTAGAAGCGGGATAAAACCACTCGCCAAGTTTTGGGTCA  < 50
CTCACAGGCACTCGCCAAGTAAACGTGGTGACTTTCTCCCGGCTCTGAGA  < 100
CAAACTTGGTGTGTGGTTTAATCCCGCTTCTAAAGTGGGCGCACGGCATG  < 150
CCAACACCGCCCCTCAGCAACTACCGTCTGCAGTCTGCCCGCGCTACACC  < 200
GTAAATTTTGGGGCGCGATACGTGTCGGTTTGGGCGGGAAAGGCGGCAGG  < 250
AAAGGCAGGCGGGAAGGGGGCATCAGTTGATGGGACTGTATGGGACTCTT  < 300
GATGGGACCGGATGGGACTTGCAATGCATTGGGCGCTGACTAACCGCACA  < 350
TCATCTGGCAATTTGTTTCCACAGTTTGTTGCGATAGAAGCGTTTCTGCG  < 400
CTGCTACGGGTGGCTTGCTGATCATATGTTGACATTTGCGGTAACTCGTT  < 450
GGCAGTCTCACGCTGCTTCTCGCGACTCTAGCGTAGTCTGATCAGTAACC  < 500
ACCTTATAGTATCCCCGCACCAGAAGTCACAGCGCATCGAGCGCCAGCAG  < 550
CTCAGGCGCGGGCTCGATGGCAACGATTGCGCGGCCTTTCGACTGTCCGC  < 600
GGTTGCCAGTCCGGCACAATGGCCTAGCCGCCACTGCATGCGCGCCAGCG  < 650
CGTATGAAGCCTGGTGTCGGAAGTGCCTCGGGCGTTCGGGCGCAGGCCGG  < 700
AGCGTCGGTCCCTGCGCCGCGATCGCCATCCCGCCCGCGCCCAGCTCGGC  < 750
GGAAGCCCGCGCCGCCGCACCCAGCAGCGGATGGCTCGGTAGCGCCACAG  < 800
CATTCGAAGGTAGGTGTGGACTACAGCTATGCGAGCTCGCGTGGCGCGAA  < 850
CGGGGATGACGTTGATGGCAACCGGCGTGCGCTCGTGACGTGACCGGTGA  < 900
GCTCCCAGCGTGGGGCCATCACAAGTGGAAGCGCCCTCCCGCTCCGCCCC  < 950
TGCACAGGCAACGCCTGCAGCGAAGGCAAGCctcgaGATGGGCGTGAAGG  < 1000
TGCTGTTCGCCCTGATCTGCATCGCCGTCGCCGAGGCGAAGCCGACGGAG  < 1050
AACAACGAGGACTTCAACATTGTCGCCGTGGCGTCCAACTTTGCCACCAC  < 1100
CGATCTGGACGCCGACCGCGGCAAGCTGCCGGGCAAGAAGCTGCCCCTGG  < 1150
AGGTCCTCAAGGAAATGGAGGCGAACGCCCGGAAGGCCGGCTGCACCCGC  < 1200
GGCTGCCTAATCTGCCTGTCCCACATCAAGTGCACGCCGAAGATGAAGAA  < 1250
GTTCATTCCCGGCCGGTGCCACACCTACGAGGGCGACAAGGAGTCCGCGC  < 1300
AGGGCGGCATTGGCGAGGCGATCGTGGACATCCCCGAGATTCCGGGCTTC  < 1350
AAGGACCTGGAGCCGATGGAGCAGTTCATCGCCCAGGTGGACCTGTGCGT  < 1400
GGACTGCACGACCGGCTGTCTGAAGGGCCTGGCCAATGTGCAGTGTTCCG  < 1450
ACCTGCTCAAGAAGTGGCTGCCCCAGCGCTGTGCCACCTTCGCCAGCAAG  < 1500
ATCCAGGGCCAGGTGGACAAGATTAAGGGCGCTGGCGGCGATACGTAAGG  < 1550
ATCCCCGCTCCGTGTAAATGGAGGCGCTCGTTGATCTGAGCCTTGCCCCC  < 1600
TGACGAACGGCGGTGGATGGAAGATACTGCTCTCAAGTGCTGAAGCGGTA  < 1650
GCTTAGCTCCCCGTTTCGTGCTGATCAGTCTTTTTCAACACGTAAAAAGC  < 1700
GGAGGAGTTTTGCAATTTTGTTGGTTGTAACGATCCTCCGTTGATTTTGG  < 1750
CCTCTTTCTCCATGG                                     < 1765
```

```
Features :
DMT4 region               : [6 : 982]
DMT4 5'-UTR               : [503 : 653]
DMT4 1st exon CDS         : [654 : 809]
ZYRE-like motif           : [886 : 894]
DMT4 2nd exon CDS         : [958 : 981]
cgLuc                     : [982 : 1543]
RBCS2 3-UTR               : [1544 : 1760]
```

ϕ-DMT4(−498 to +479) *::cgLuc*; **Error! Reference source not found.**

```
TctagaGTGAGCTAGAAGCGGGATAAAACCACTCGCCAAGTTTTGGGTCA   < 50
CTCACAGGCACTCGCCAAGTAAACGTGGTGACTTTCTCCCGGCTCTGAGA   < 100
CAAACTTGGTGTGTGGTTTAATCCCGCTTCTAAAGTGGGCGCACGGCATG   < 150
CCAACACCGCCCCTCAGCAACTACCGTCTGCAGTCTGCCCGCGCTACACC   < 200
GTAAATTTTGGGGCGCGATACGTGTCGGTTTGGGCGGGAAAGGCGGCAGG   < 250
AAAGGCAGGCGGGAAGGGGGCATCAGTTGATGGGACTGTATGGGACTCTT   < 300
GATGGGACCGGATGGGACTTGCAATGCATTGGGCGCTGACTAACCGCACA   < 350
TCATCTGGCAATTTGTTTCCACAGTTTGTTGCGATAGAAGCGTTTCTGCG   < 400
CTGCTACGGGTGGCTTGCTGATCATATGTTGACATTTGCGGTAACTCGTT   < 450
GGCAGTCTCACGCTGCTTCTCGCGACTCTAGCGTAGTCTGATCAGTAACC   < 500
ACCTTATAGTATCCCCGCACCAGAAGTCACAGCGCATCGAGCGCCAGCAG   < 550
CTCAGGCGCGGGCTCGATGGCAACGATTGCGCGGCCTTTCGACTGTCCGC   < 600
GGTTGCCAGTCCGGCACAATGGCCTAGCCGCCACTGCATGCGCGCCAGCG   < 650
CGTATGAAGCCTGGTGTCGGAAGTGCCTCGGGCGTTCGGGCGCAGGCCGG   < 700
AGCGTCGGTCCCTGCGCCGCGATCGCCATCCCGCCCGCGCCCAGCTCGGC   < 750
GGAAGCCCGCGCCGCCGCACCCAGCAGCGGATGGCTCGGTAGCGCCACAG   < 800
CATTCGAAGGTAGGTGTGGACTACAGCTATGCGAGCTCGCGTGGCGCGAA   < 850
CGGGGATGACGTTGATGGCAACCGGCGTGCGCTCGTAACGTTACCGGTGA   < 900
GCTCCCAGCGTGGGGCCATCACAAGTGGAAGCGCCCTCCCGCTCCGCCCC   < 950
TGCACAGGCAACGCCTGCAGCGAAGGCAAGCctcgaGATGGGCGTGAAGG   < 1000
TGCTGTTCGCCCTGATCTGCATCGCCGTCGCCGAGGCGAAGCCGACGGAG   < 1050
AACAACGAGGACTTCAACATTGTCGCCGTGGCGTCCAACTTTGCCACCAC   < 1100
CGATCTGGACGCCGACCGCGGCAAGCTGCCGGGCAAGAAGCTGCCCCTGG   < 1150
AGGTCCTCAAGGAAATGGAGGCGAACGCCCGGAAGGCCGGCTGCACCCGC   < 1200
GGCTGCCTAATCTGCCTGTCCCACATCAAGTGCACGCCGAAGATGAAGAA   < 1250
GTTCATTCCCGGCCGGTGCCACACCTACGAGGGCGACAAGGAGTCCGCGC   < 1300
AGGGCGGCATTGGCGAGGCGATCGTGGACATCCCCGAGATTCCGGGCTTC   < 1350
AAGGACCTGGAGCCGATGGAGCAGTTCATCGCCCAGGTGGACCTGTGCGT   < 1400
GGACTGCACGACCGGCTGTCTGAAGGGCCTGGCCAATGTGCAGTGTTCCG   < 1450
ACCTGCTCAAGAAGTGGCTGCCCCAGCGCTGTGCCACCTTCGCCAGCAAG   < 1500
ATCCAGGGCCAGGTGGACAAGATTAAGGGCGCTGGCGGCGATACGTAAGG   < 1550
ATCCCCGCTCCGTGTAAATGGAGGCGCTCGTTGATCTGAGCCTTGCCCCC   < 1600
TGACGAACGGCGGTGGATGGAAGATACTGCTCTCAAGTGCTGAAGCGGTA   < 1650
GCTTAGCTCCCCGTTTCGTGCTGATCAGTCTTTTTCAACACGTAAAAAGC   < 1700
GGAGGAGTTTTGCAATTTTGTTGGTTGTAACGATCCTCCGTTGATTTTGG   < 1750
CCTCTTTCTCCATGG                                      < 1765
```

```
Features :
DMT4 region            : [6 : 982]
DMT4 5'-UTR            : [503 : 653]
DMT4 1st exon CDS      : [654 : 809]
Mutated ZYRE-like motif : [886 : 894]
DMT4 2nd exon CDS      : [958 : 981]
cgLuc                  : [982 : 1543]
RBCS2 3-UTR            : [1544 : 1760]
```

Figure S1. Sequences of transgenes used in the luciferase expression experiments.
