# Supplementary material for: Identification and Characterization of a cis-Regulatory Element for Zygotic Gene Expression in Chlamydomonas reinhardtii
Source: G3 (Bethesda). 2016 Mar 23;6(6):1541–8. doi: 10.1534/g3.116.029181 (PMC4889651; doi:10.1534/g3.116.029181)
Supplement: Supplemental Material [file supp_g3.116.029181_TableS2.pdf]

**Table S2.** Primers used in this study.

| Name | Sequence |
|---|---|
| gsp01 | TATGAGCTTGTGCGCGATAC |
| gsp02 | CTGTTGGTGGTGTTGGTGAG |
| oMJ155 | GCTCGTGGAGCTCTGAATCT |
| GSP1-3fwd | TACCTTGTCTCAGCCCTATACT |
| GSP1-3rev | CTCAAGGCGGATGCGATATTA |
| 7_bHLH_F | TGACTTCCTTGGCGGAGATGACAA |
| 7_bHLH_R | TGGACGTCAAAGCTGGTATGGTGA |
| ZYS3p-XbaI-F | ctctagaGCAGTCGGGTCGTGTCGCCT |
| ZYS3-nomotif-F | ctctagaTGGCTGTAGCGCCCGGCCCGCAT |
| ZYS3-motif-5UTR-F | ctctagaGCAGTCGGGTCGTGTCGCCTCGATTCGACACGACC TTCTTCCTCTCGCTTAC |
| ZYS3-5UTR-F | ctctagaaCTTCTTCCTCTCGCTTACCT |
| ZYS3-Z-nomotif-F | ctctagaGGTGACATGACTGGCTGTAGCGCCCGGCCCGCAT |
| ZYS3UP-XhoI-REV | ggggctcgagCGAGGGATGTGTTCGGTGGA |
| TM-XbaI-NdeI-F | ctctagacatatgCTGTTTAAATAGCCAGG |
| TM-XbaI-A-F | ctctagaGCAGTCGGGTCGTGTCGCCTCGATTCGACACGACC TGTTTAAATAGCCAGG |
| TM-SalI-R | cccgtcgacGTTTGCGGGTTGTGACTGA |
| CMT1UP-XbaI-FWD | ccctctagaTGCCGTGAGCTAGAAGCGGG |
| CMT1UP-XhoI-REV | ggggctcgagGCTTGCCTTCGCTGCAGGCG |
| DMT4-sub-ZYRE-F | ACCGGCGTGCGCTCGT**a**ACGT**t**ACCGGTGAGCTCCCAGCGTG |
| DMT4-sub-ZYRE-R | CACGCTGGGAGCTCACCGGT**a**ACGT**t**ACGAGCGCACGCCGGT |
| Cgluc-upXbaI | TCAAGACCCGTTTAGAGG |
| Glucrev4 | GCGACAATGTTGAAGTCCTCGTT |
