# Supplementary material for: Identification and Characterization of a cis-Regulatory Element for Zygotic Gene Expression in Chlamydomonas reinhardtii
Source: G3 (Bethesda). 2016 Mar 23;6(6):1541–8. doi: 10.1534/g3.116.029181 (PMC4889651; doi:10.1534/g3.116.029181)
Supplement: Supplemental Material [file supp_g3.116.029181_TableS4.pdf]

Table S4. MEME results.

Motif number	Motif Logo	*E*-value	Sites
#1	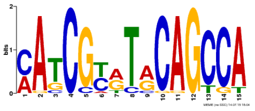	2.4e-024	31
#2	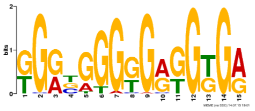	2.9e-024	>50 (MEME count limit)
#3 (ZYRE)	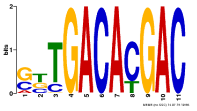	1.4e-024	157 (FIMO count)
#4	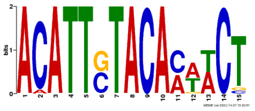	1.9e-021	16
#5	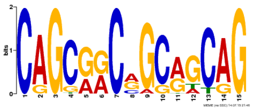	3.8e-015	31
#6	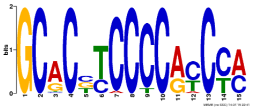	2.5e-015	27
#7	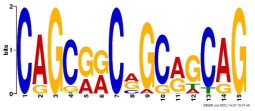	7.6e-018	21
#8	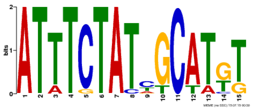	3.2e-011	14
#9	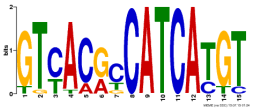	4.3e-010	16
#10	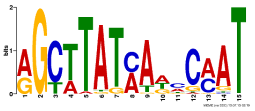	1.1e-012	31
